# LeishVet update and recommendations on feline leishmaniosis

**DOI:** 10.1186/s13071-015-0909-z

**Published:** 2015-06-04

**Authors:** Maria-Grazia Pennisi, Luís Cardoso, Gad Baneth, Patrick Bourdeau, Alek Koutinas, Guadalupe Miró, Gaetano Oliva, Laia Solano-Gallego

**Affiliations:** Department of Veterinary Sciences, University of Messina, Polo Universitario Annunziata, Messina, 98168 Italy; Department of Veterinary Sciences, School of Agrarian and Veterinary Sciences, University of Trás-os-Montes e Alto Douro (UTAD), Vila Real, 5000-801 Portugal; Koret School of Veterinary Medicine, The Hebrew University of Jerusalem, P.O. Box 12, Rehovot, 76100 Israel; Veterinary School of Nantes ONIRIS, University of Nantes, LUNAM, Nantes, 44307 France; Quality Vet-Practice, Volos, Greece; Department of Animal Health, Veterinary Faculty, Universidad Complutense de Madrid, Madrid, Spain; Department of Veterinary Medicine and Food Production, University of Naples “Federico II”, Via Delpino 1, Naples, 80137 Italy; Departament de Medicina i Cirurgia Animal, Facultat de Veterinaria, Universitat Autonoma de Barcelona, Bellaterra, 08193 Spain

**Keywords:** Feline leishmaniosis, *Leishmania infantum*, Epidemiology, Diagnosis, Treatment, Prognosis, Prevention, Recommendations

## Abstract

Limited data is available on feline leishmaniosis (FeL) caused by *Leishmania infantum* worldwide. The LeishVet group presents in this report a review of the current knowledge on FeL, the epidemiological role of the cat in *L. infantum* infection, clinical manifestations, and recommendations on diagnosis, treatment and monitoring, prognosis and prevention of infection, in order to standardize the management of this disease in cats. The consensus of opinions and recommendations was formulated by combining a comprehensive review of evidence-based studies and case reports, clinical experience and critical consensus discussions. While subclinical feline infections are common in areas endemic for canine leishmaniosis, clinical illness due to *L. infantum* in cats is rare. The prevalence rates of feline infection with *L. infantum* in serological or molecular-based surveys range from 0 % to more than 60 %. Cats are able to infect sand flies and, therefore, they may act as a secondary reservoir, with dogs being the primary natural reservoir. The most common clinical signs and clinicopathological abnormalities compatible with FeL include lymph node enlargement and skin lesions such as ulcerative, exfoliative, crusting or nodular dermatitis (mainly on the head or distal limbs), ocular lesions (mainly uveitis), feline chronic gingivostomatitis syndrome, mucocutaneous ulcerative or nodular lesions, hypergammaglobulinaemia and mild normocytic normochromic anaemia. Clinical illness is frequently associated with impaired immunocompetence, as in case of retroviral coinfections or immunosuppressive therapy. Diagnosis is based on serology, polymerase chain reaction (PCR), cytology, histology, immunohistochemistry (IHC) or culture. If serological testing is negative or low positive in a cat with clinical signs compatible with FeL, the diagnosis of leishmaniosis should not be excluded and additional diagnostic methods (cytology, histology with IHC, PCR, culture) should be employed. The most common treatment used is allopurinol. Meglumine antimoniate has been administered in very few reported cases. Both drugs are administered alone and most cats recover clinically after therapy. Follow-up of treated cats with routine laboratory tests, serology and PCR is essential for prevention of clinical relapses. Specific preventative measures for this infection in cats are currently not available.

## Introduction and history of feline leishmaniosis

*Leishmania infantum* (syn. *Leishmania chagasi*) infection is found both in the Old and New Worlds with dogs as the main reservoir. Canine leishmaniosis (CanL) is an important and complex zoonotic disease whose transmission, pathogenesis, clinical manifestations, diagnosis, therapy and prevention have been extensively studied [1, 2]. Conversely, in the last century, the cat was usually considered as a relatively resistant host species to *Leishmania* infection based on two experimental studies (see Question 5) and on limited numbers of clinical case reports and histopathological descriptions of the presence of *Leishmania* infection in necropsies.

Historically, some studies have used cats for investigating their potential role as reservoir for *Leishmania*. Pet cats living in the same houses where human cases of cutaneous or visceral leishmaniosis were diagnosed were examined for the presence of *Leishmania* amastigotes in skin lesions or by *post mortem* histopathological evaluation of the bone marrow and spleen [3, 4]. In Sicily (southern Italy), no case of infection was found by cytological and histological examination of spleen, liver and bone marrow of 120 necropsied cats living in an endemic area [5]. The same negative results were obtained in Egypt when spleen cytology and culture were performed on 28 stray cats, and six of them displaying skin lesions were negative also from skin [6]. Conversely, in Jordan, amastigotes were detected in liver and spleen smears from about 20 % of 78 stray cats [7].

The development of both feline medicine and more sensitive and specific diagnostic techniques such as serological and molecular methods has led in recent decades to an increasing number of documented case reports of feline leishmaniosis (FeL) and subclinical infections. However, there is still limited information on epidemiological and clinical aspects of *Leishmania* infection in cats which is all derived from descriptive studies, case reports, information from canine leishmaniosis cases and personal experience of respected experts. This means that the current quality of evidence supporting any recommendation on feline leishmaniosis is low (grade IV) [8].

In this report the LeishVet group presents an overview on current knowledge on *Leishmania* infection in cats. Moreover, recommendations on the diagnosis, treatment and monitoring, prognosis and prevention of FeL are also described in order to standardize the management of this infection in cats. These were constructed by combining a comprehensive review of evidence-based studies and case reports, clinical experience and critical consensus discussions. The goal of this review is therefore to offer the veterinary practitioners an updated approach with recommendations on the management of leishmaniosis in cats.

## Review

### Etiology and transmission

1. What species of *Leishmania* infect cats? What is their geographical distribution?

Five species within the genus *Leishmania* have been identified in cats: *Leishmania mexicana*, *Leishmania venezuelensis, Leishmania braziliensis* and *Leishmania amazonensis* in the New World, and *Leishmania infantum* in both the New and Old Worlds (Table 1). We can therefore state that cats are likely to be infected by the same *Leishmania* species found in humans or other animals in the same geographic area.

**Table 1:** Species of Leishmania identified in cats and geographical areas of description

Species	Country (area)	Method	Global distribution	Reference
*Leishmania amazonensis*	Brazil (Mato Grosso do Sul state)	ILMA	South America	[73]
*Leishmania braziliensis*	Brazil (Belo Horizonte city)	PCR and hybridization	Central and South America	[92]^c^
	Brazil (Rio de Janeiro city)	MLEE		[74]
	France (French Guiana)	PCR and sequencing		[93]^d^
*Leishmania infantum*	Iran (Fars and East Azerbaijan provinces)	PCR and MLEE	China, Middle East, Mediterranean basin, and Central and South America	[94]
	Italy (Imperia, Liguria)	PCR-RFLP		[26]
	Italy (Messina, Sicily)	MLEE		[10]
	Italy (Lipari island, Sicily)	MLEE and PCR-RFLP		[11]
	Switzerland^a^	PCR and sequencing		[50]
	France (Alpes-Maritimes)	MLEE		[12]
	Spain (Barcelona)	PCR and sequencing		[29]
	Spain (Madrid community)	PCR and sequencing		[33, 78]
	Spain^a^	ILMA		[68]
	Spain (Mallorca)^b^	PCR-RFLP		[95]
	Portugal (Lisbon region)	PCR and sequencing		[96, 97]
	Portugal (Lisbon and Algarve regions)	PCR and sequencing		[98]
	Greece (Thessaly and Macedonia)	PCR and sequencing		[99]
	Brazil (Cotia, São Paulo state)	PCR and sequencing		[100]
	Brazil (Rio de Janeiro)	PCR and hybridization		[101]
	Brazil (Andradina, São Paulo state)	PCR and sequencing		[102, 103]
	Brazil (Araçatuba, São Paulo state)	PCR and sequencing		[104]
*Leishmania mexicana*	USA (Texas)	MLEE	North and Central America	[75]^e^
	USA (Texas)	PCR and sequencing		[76]
*Leishmania venezuelensis*	Venezuela (Barquisimeto city)	MLEE and ILMA	South America	[105]

^a^No data available on the exact origin; ^b^Feral cats; ^c^Subgenus *Viannia* (species *L. braziliensis* geographically assumed); ^d^ *L. braziliensis* complex (species *L. braziliensis* reasonably assumed; *Leishmania peruviana* species geographically excluded); ^e^ *L. mexicana* complex; ILMA: immunolabelling with monoclonal antibodies; MLEE: multilocus isoenzyme electrophoresis; PCR: polymerase chain reaction; RFLP: restriction fragment length polymorphism

Species, strains, isolates and genetic variants of *Leishmania* spp. found in cats have been characterized by means of laboratory procedures including electrophoresis of isoenzymes upon parasite cultivation, monoclonal antibodies and molecular methods. The latter mainly comprise conventional and real time polymerase chain reaction (PCR) combined with DNA sequence analysis, restriction fragment length polymorphism (RFLP) or hybridization of amplified products with specific probes (Table 1).

In southern European countries, canine and human leishmaniosis are mainly caused by *L. infantum* zymodeme MON-1 [9]. This occurs also in FeL [10–14], but zymodemes MON-72 and MON-201 have also been isolated in two single cases from Sicily [10].

2. How is *Leishmania* transmitted to the cat?

There is no specific information on the transmission of *Leishmania* spp. to cats. However, due to the extensive data on vectorial transmission of the *Leishmania* group of protozoal parasites to vertebrates, there is no doubt that the essential mode of transmission is by bites of infectious phlebotomine sand flies as for other vertebrate species. This means that in areas where *L. infantum* is transmitted to dogs, cats are likely to be in contact with the parasite and can also be potentially infected. The sand fly vectors appear to be more permissive in their blood source preferences than thought before. Several studies have demonstrated that cats constitute sources of blood for sand flies [15–19]. Moreover, the experimental demonstration of infectiousness of two infected cats to sand flies [11, 20] indirectly proves the ability of the vector to properly complete feeding on cats and acquire infection.

To date, other routes of transmission including vertical or horizontal pathways have not been described or demonstrated in cats as they have been in dogs, mice or humans [1].

### Epidemiology including risk factors and geographical distribution

3. What is the prevalence of *L. infantum* infection in endemic regions?

The prevalence of *L. infantum* infection in cat populations is commonly estimated by detection of specific antibodies, and DNA amplification by PCR [21]. Over the last few decades, many studies have confirmed that feline *Leishmania* infection may be relatively common in areas where CanL is endemic. Seroprevalence ranges from 0 to 68.5 % and molecular rates of infection range from 0 and 60.7 % in endemic regions of the Old World (Table 2). Therefore, a high variability in antibody or molecular prevalences is evident from published investigations, and this may be due to different levels of endemicity, characteristics of the population under study or differences in diagnostic methodologies including the cut-off titres of serology. Moreover, few studies validated the serological techniques in cats by using feline positive control sera obtained from cats with clinical illness confirmed by isolation and negative control sera from a substantial number of cats from non endemic areas [22–25].

**Table 2:** Prevalence of Leishmania infantum infection in cats in the Old World (countries listed in geographical order from East to West)

Country (area)	No. of cats (type)	Seroprevalence (test)	PCR prevalence (sample)	Combined prevalence of infection	Prevalence of clinical signs in positive cats	Reference
Iran (Fars and East Azerbaijan provinces)	40 (stray)	27.5 % (IFAT and DAT)	NA	NA	NA	[27]
Iran (Fars and East Azerbaijan provinces)	40 (stray)	NA	7.5 % (liver and spleen)	10.0%^a^	25.0 % (cutaneous)	[94]
Israel (Jerusalem)	104 (mainly stray)	6.7 % (ELISA)	NA	NA	14.3%^b^ (cutaneous)	[30]
Egypt (Ismailia governorate)	80 (stray)	3.8 % (IHAT)	NA	NA	NA	[106]
Egypt (Suez governorate)	28 (stray)	3.6 % (IHAT)	NA	NA	NA	[6]
Egypt (Giza governorate)	60 (mixed)	10.0 % (IHAT)	NA	NA	NA	[4]
Greece (Thessaloniki)	284 (stray)	3.9 % (ELISA)	NA	NA	0.0 %	[107]
Greece (Thessaloniki)	389 (stray/feral)	21.6 % (IFAT)	NA	NA	19.0 % (compatible)	[108]
Greece (Macedonia and Thessaly)	100 (domestic)	11.0 % (IFAT and ELISA)	41.0 % (skin, bone marrow, blood and conjunctiva)	46.0 %^c^	39.1 % (cutaneous, ocular or systemic)	[88, 99]
Albania (Tirana surroundings)	146 (stray)	0.7 % (IFAT)	0.0 % (blood)	0.7 %	0.0 %	[109]
Italy (Sicily)	93 (mixed)	59.1 % (IFAT)	NA	NA	0.0 % (cutaneous)	[35]
Italy (Catania and Messina provinces, Sicily)	89 (mixed)	68.5 % (IFAT)	60.7 % (blood)	85.4 %	NA^b^	[32]
Italy (Liguria and Tuscany)	110 (domestic)	0.9 % (IFAT)	NA	NA	0.0 %	[26]
Italy (Abruzzo)	203 (mixed)	16.3 % (IFAT)	45.5 (blood), 100 % (lymph node)^d^	NA	66.4 % (heterogeneous)	[110]
Italy (Ischia island, Campania)	95 (mixed)	9.5 % (IFAT)	5.3 (blood), 0.0 % (bone marrow)	13.7 %	0.0 %	[77]
Italy (Calabria and Sicily)	431 (mixed)	6.9 % (IFAT)	7.8 % (blood), 11.7 % (lymph node), 16.7 % (conjunctival swabs)	13.9 %	NA^e^	[24]
Italy (Greater Milan)	233 (stray)	25.3 % (IFAT)	0.0 % (blood)	25.3 %	79.7 % (heterogeneous)^b^	[111]
France (Nice surroundings)	97 (stray)	12.4 % (WB)	NA	NA	0.0 %	[12]
Spain (Barcelona and Girona provinces)	117 (domestic)	1.7 % (ELISA)	NA	NA	NA	[112]
Spain (Aragon)	50 (domestic)	42.0 % (DAT)	NA	NA	100 % (immune dysfunction)	[113]
Spain (Catalonia and Mallorca island)	445 (mixed)	5.3–6.3 % (ELISA)^f^	NA	NA	NA^b^	[22]
Spain (south)	183 (domestic)	28.3–60.0 % (IFAT)^f^	25.7 % (blood)	70.6 %	NA	[114]
Spain (Barcelona)	100 (domestic)	NA	3.0 % (blood)	NA	100 % (ND)	[29]
Spain (Madrid community)	233 (domestic)	1.3–4.3 % (IFAT)^f^	0.4 % (blood)	1.7–4.7%^f^	66.7 % (heterogeneous)^b^	[78]
Spain (Ibiza island)	105 (stray/shelter)	13.2 % (ELISA)	8.7 % (blood)	15.4 %	25.0 % (cutaneous)^g^	[25]
Spain (Mallorca island)	86 (stray/feral)	15.7 % (WB)	26.0 % (blood)	25.6 %	0.0 %	[95]
Spain (Madrid community)	20 (breeding cats)	15.0 % (IFAT)	NA	NA	0.0 %	[115]
Spain (Madrid community)	680 (mixed)	3.7 % (IFAT)	0.6 % (blood)	NA	NA^e^	[33]
Spain (Madrid community, Guadalajara and Toledo provinces)	346 (stray)	3.2 % (IFAT)	0.0 % (blood)	3.2 %	9.1 % (compatible)^b^	[34]
Portugal (Lisbon region)	23 (stray)	20.0 % (IFAT)	30.4 % (blood)	34.8 %	0.0 % (compatible)	[96]
Portugal (northeast)	316 (domestic)	2.8 % (ELISA and DAT)	NA	NA	11.1 % (ND)^b^	[23]
Portugal (Lisbon region)	180 (stray)	0.6 % (IFAT)	NA	NA	0.0 % (compatible)	[116]
Portugal (Lisbon region)	142 (domestic)	1.3 % (IFAT)	20.3 % (blood)	20.4 %	NA	[97]
Portugal (North and Centre regions)	320 (domestic)	NA	0.3 % (blood)	NA	0.0%^b^	[117]
Portugal (Lisbon and Algarve regions)	649 (mixed)	NA	9.9 % (blood)	NA	27.3 % (compatible)^b^	[98]
Portugal (Algarve)	271 (mixed)	3.7 % (DAT)	NA	NA	NA	[118]

^a^PCR results in combination with those from liver and spleen touch smears and cultures; ^b^No statistical association between clinical status and prevalence of infection/exposure; ^c^Negative results of lymph node, bone marrow, skin and conjunctiva cytology; ^d^PCR performed only for 11 seropositive cats; ^e^Statistical association between clinical status and both seroprevalence and combined prevalence; ^f^Different prevalences obtained with different ELISA techniques or IFAT cut off; ^g^Statistical association between clinical status and seroprevalence; DAT: direct agglutination test (cut-off titre: 1:100 or 1:800); ELISA: enzyme-linked immunosorbent assay (different techniques); IFAT: immunofluorescence antibody test (cut-off titre ranging from 1:2 to 1:100); IHAT: indirect haemagglutination test (cut-off titre: 1:32); NA: not assessed/available; ND: clinical signs not described; PCR: polymerase chain reaction; WB: western blot

However, it is important to highlight that clinical illness and subclinical infection in cats are less frequently reported than in their canine counterparts. In fact, the seroprevalence of *Leishmania* infection in cats is lower than in dogs from the same locations [23, 26–28] and a lower PCR prevalence in cats than in dogs is also reported from similar geographical areas [29]. Immune responses leading to natural feline resistance might account for the observed differences in the prevalence of infection in cats as compared to dogs. Studies evaluating *Leishmania* specific cellular immunity tests in cats could better estimate infection, but they are still lacking in cats [22].

Limited epidemiological studies have reported significant association between *L. infantum* infection diagnosed by serology or PCR and seasonality [24], altitude [30], rural habitat [23], outdoor lifestyle [12], male gender [23, 31, 32] and adult age [23, 24, 32, 33]. Feline *L. infantum* coinfections with feline leukemia virus (FeLV), feline immunodeficiency virus (FIV), feline coronavirus (FCoV) and/or *Toxoplasma gondii* have been reported in the literature [24, 25, 31, 33–37], but a significant association was found only between *L. infantum* positivity (molecular or serological) and FIV [25, 33, 35].

4. What is the epidemiological role of *L. infantum* infected cats?

Domestic dogs are considered the only known primary reservoir for *L. infantum* infection [38]. It has been considered for a long time that cats did not play any role in the epidemiology of *L. infantum* in endemic areas. This view was directed by the facts that, for a long period, very few cases of clinical leishmaniosis were described in cats as compared to dogs, and that cats have also been considered more resistant to experimental infection [39].

This interpretation has changed, as the concepts of reservoir and susceptibility in infected hosts are now better understood. The majority of infected dogs does not exhibit clinical signs (at least for a long period), although they can be infectious to sand flies and consequently serve as sources of infection. During the last two decades, many wild mammals have been diagnosed with *Leishmania* infection by serological and/or molecular methods [40]. However, their role as reliable sources of infection (infectiousness to sand flies, persistent infection) remains unknown [40]. The recent demonstration that hares can be persistently infected, infectious to sandflies and a reservoir for humans in the absence of participation of dogs in the transmission cycle opens a possible evaluation of the role of species other than dogs in the epidemiology of *L. infantum* infections in particular scenarios [41, 42].

Surveys have shown that the percentage of infected cats is not negligible in some endemic areas (Table 2). In cats, disease and infection may persist for very long periods and cats have been shown to be infectious to sand flies in experimental xenodiagnosis studies both in the Old and New Worlds. They may, therefore, play some role in the transmission of *L. infantum* in regions where many cats are infected [43].

In cats, infection could be promoted by concurrent immunosuppressive infections such as FIV or FeLV [13, 31]. The fact that cats appear to better control the infection and more rarely manifest the disease is also in favour of a potential persistent source role of infected individuals. Moreover, the population of pet and stray cats may be even larger than that of dogs in some endemic areas [44].

According to the current state of the art, cats are most likely a secondary reservoir of *L. infantum* which will not support persisting infection in a natural setting if the primary reservoir is absent, e.g. cats alone would not be responsible for the persistence of *L. infantum* infection in an area where disease transmission is possible with abundant competent sand fly vectors, unless infected dogs are present. The epidemiological role of cats in the maintenance and transmission of *L. infantum* should nevertheless be further investigated [43]. Questions that need to be addressed include: 1) are cats involved in the transmission of parasite by sand fly vectors in endemic areas where both infected cats and dogs are present? 2) how attractive are cats to vector sandflies? 3) how accessible is the parasite in infected cats to sand flies?

### Experimental *Leishmania* infection

5. What is known about experimental *Leishmania* infection in cats?

Reports on experimental feline infections with *Leishmania* spp. are very scarce. Only two studies have been reported with different species of the *Leishmania donovani* complex and were both performed many years ago [39, 45]. This means that sensitive techniques such as PCR were not applied for monitoring infected cats. A third study was conducted more recently in Brazil with *L. braziliensis* [46]. Parasitological, serological and clinical details on the experimental studies carried out in cats are shown in Table 3.

**Table 3:** Parasitological, serological, and clinical results from experimental Leishmania infections in cats

Cats (n)	*Leishmania* species	Inoculum	Route	Sampling	Evidence of infection	Serology	Clinical abnormalities	Reference
10	*L. infantum* (French strain)	8 × 10^8^ amastigotes (isolated from a French dog and maintained by serial passages in golden hamsters)	IV	2 cats necropsied at 1 h PI, and weeks 1, 2, 4 and 8 PI	Parasites in spleen, liver, bone marrow (cytology or culture) and blood culture from 1 to 8 weeks PI	IFAT: highly positive from 1 to 8 weeks PI	None	[39]
5	*L. infantum* (Brazilian strain)	5 × 10^7^ amastigotes (isolated from a human being in Brazil and maintained by serial passages in golden hamsters)	IV	Cats necropsied at weeks 4 (*n* = 1), 16 (*n* = 2) and 24 (*n* = 2)	Parasites in spleen, liver and bone marrow (cytology or culture) from week 4 to 16, but not at week 24 (no parasites cultured from blood at any point)	IFAT: highly positive from weeks 2 to 24 (rise to 30-fold at the end of the study)	None	[39]
6	*L. infantum* (Brazilian strain)	5 × 10^7^ promastigotes (*in vitro* cultivation of the above human strain)	ID (thorax)	Pairs of cats necropsies at weeks 4, 16 and 24; blood culture at weeks 2, 4, 8, 12, 16 and 24	No parasites detected at necropsy (bone marrow, spleen or liver) or blood cultures	IFAT: positive from weeks 2 to 24 (lower than for cats IV inoculated)	None	[39]
3	*L. infantum* (Brazilian strain)	10^8^ promastigotes (derived from cultures of the splenic tissue from one IV inoculated cat)	ID	Necropsies at 12 weeks PI	Negative cultures of different tissues	ND	None	[39]
6 + 6	*L. donovani* (Kenyan strain)	10^6^ promastigotes	IC + IV	2 cats necropsied at months 1 to 6 PI	Negative on blood, bone marrow, liver, spleen, kidney and lymph node cultures and smears	ND	None	[45]
13	*L. braziliensis* (Brazilian strain)	10^7^ promastigotes	ID (ear and nose)	Follow-up for 72 weeks (*n* = 9)	Positive parasite cultures from aspirates of a primary ear lesion at week 6	ELISA: positive at week 2; all cats were seropositive at week 20; after self-healing, 3 cats remained seropositive until the end of the study and none of them had lesion recurrence	Single papules on the ear and nose as early at week 2; regression at about 32 and 40 weeks PI in the ear and nose, respectively; one cat had lesion recurrence on the ear 4 months after self-healing	[46]
				4 cats necropsied at weeks 4, 12, 16 and 24	Negative cultures and imprints from liver, spleen and bone marrow			

ELISA: enzyme-linked immunosorbent assay; IC: intracardiac; ID: intradermal; IFAT: immunofluorescence antibody test; IV: intravenous; ND: not done; PI: post-infection

Based on these studies, cats are apparently less susceptible than dogs [47, 48] to the development of disease after established experimental infection with species of the *L. donovani* complex or are even resistant to infection [15].

In contrast after experimental infection with *L. braziliensis*, domestic cats develop self-healing chronic cutaneous lesions containing parasites as often seen in dogs [49].

### Clinical presentation

6. What are the most common clinical findings of FeL due to *L. infantum*?

Detailed case reports of FeL have been available in recent years mainly from European countries where pet cats typically have a higher standard of health care. In the New World, other *Leishmania* spp. are endemic and may co-infect cats and complicate the clinical picture [28]. Therefore, we have only reviewed case reports or case series originally from European countries. A total of 46 clinical cases have been published between 1989 and 2014, where the diagnosis of FeL was confirmed by serological and/or parasitological methods [11–14, 21, 26, 36, 37, 50–67].

The most common clinical signs reported in FeL include skin or mucocutaneous lesions and lymph node enlargement, and they have been described in more than half of the cases (Table 4). Some cats showed only dermatological lesions alone [13, 52, 56, 58], while others with skin lesions showed a combination with systemic signs [12, 14, 21, 26, 36, 51, 60, 62–64, 68]. Conversely, other cats did not have any skin detectable lesions on clinical presentation [11, 36, 50, 54, 55, 57, 66, 69, 70].

**Table 4:** Frequency (%) of clinical manifestations described in a total of 46 case reports and 15 histopathological case descriptions of feline leishmaniosis from European countries (1989–2014)

Frequency of clinical manifestations (%)		
~50 %	20–30 %	<10 %
Lymph node enlargement	Ocular lesions (mainly uveitis)	Pale mucous membranes
Skin and/or mucocutaneous lesions (mainly ulcerative or nodular)	Oral lesions	Hepatomegaly
	Weight loss	Icterus
	Anorexia	Cachexia
	Lethargy	Fever
	Dehydration	Vomiting
		Diarrhea
		Chronic nasal discharge
		Splenomegaly
		Polyuria/polydipsia
		Itching
		Dyspnea
		Wheezing
		Abortion
		Hypothermia

**Fig. 1:**
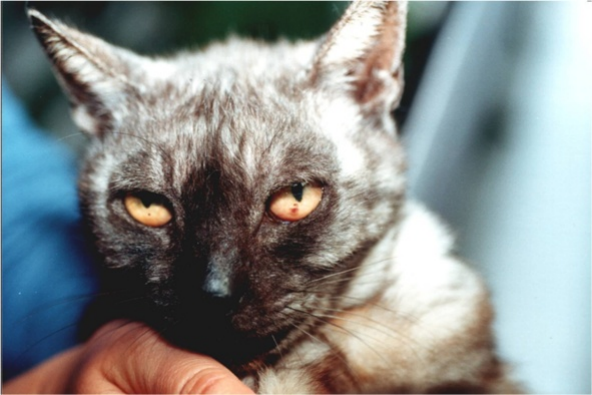
Clinical findings of feline leishmaniosis due to *Leishmania infantum*: bilateral uveitis with blood clot (hyphema) in the anterior chamber

**Fig. 2:**
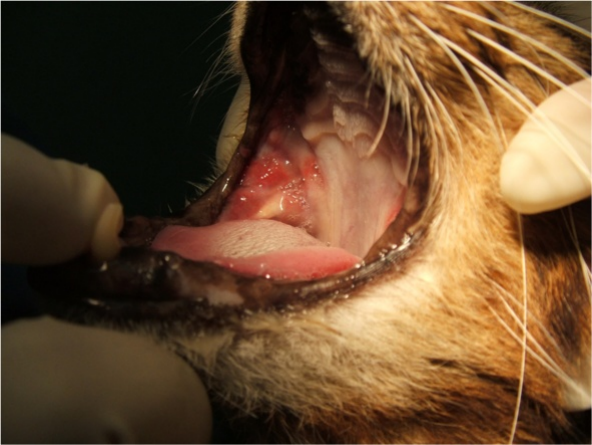
Clinical findings of feline leishmaniosis due to *Leishmania infantum*: stomatitis and glossitis involving respectively cheeks and margin of the tongue

The cutaneous and mucocutaneous lesions are described in Question 7. Lymphadenomegaly may be solitary or multicentric. Ocular lesions have been reported in approximately one third of the affected cats. Uveitis, either unilateral or bilateral (Fig. 1), is the most common ocular lesion described, with occasionally a pseudotumoral granulomatous pattern and eventually progress to panophthalmitis [50, 53, 55, 64, 69]. Blepharitis and conjunctivitis have also been described in a number of clinical cases [66, 68, 70]. Amastigotes have been found by cytology in conjunctival nodules, corneal infiltrates and aqueous humor, and by histopathology after enucleation of the eye or *post mortem* even in uveal tissue [50, 53, 55, 64, 69]. Chronic gingivostomatitis is also a common clinical finding and has been found in about one fourth of the cats so far studied with leishmaniosis (Fig. 2) [11, 26, 53, 55, 63, 66, 70]. Nodular lesions are unfrequently seen on the gingival mucosa or the tongue [60, 66, 69, 71], where infected macrophages may be visualized in lesion biopses [60, 69].

Non specific signs such as weight loss, reduced appetite, dehydration, and lethargy also have been reported. A list of other sporadic clinical manifestations described includes: pale mucous membranes, hepatomegaly, jaundice, cachexia, fever, vomiting, diarrhea, chronic nasal discharge, splenomegaly, polyuria/polydipsia, dyspnea, wheezing, abortion and hypothermia.

The implication of *Leishmania* as a cause of some of these clinical signs has been associated with the presence of the parasite in cytological or histopathological examinations of liver, spleen, lymph nodes, stomach, large bowel, kidney, oral mucosa, nasal exudate and eye tissues [13, 14, 36, 50, 57, 63, 66, 68, 72]. However, clinical disease is commonly associated with an impaired immunocompetence due to several causes including retroviral infections (FIV and FeLV), immunosuppressive treatment and concomitant debilitating diseases such as malignant neoplasia or diabetes mellitus [44].

As also found in dogs, FeL does not exclude the possibility of concurrent diseases or co-infections. This fact may influence the clinical presentation and prognosis. The cause-effect relationship between various etiological and pathogenic factors is not always easy to establish [21].

7. What are the most common dermatological findings of FeL due to *L. infantum* and to other *Leishmania* species?

**Fig. 3:**
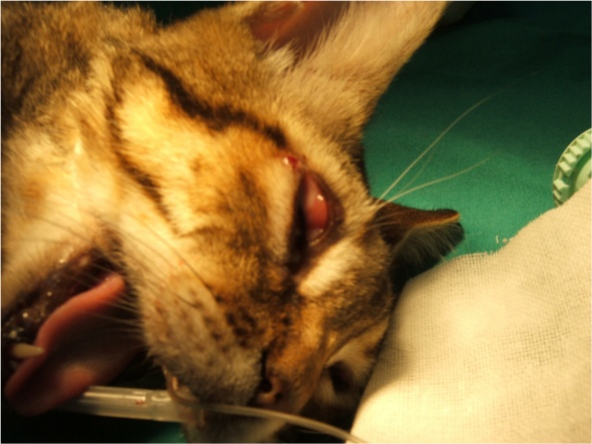
Clinical findings of feline leishmaniosis due to *Leishmania infantum*: nodular conjunctivitis (upper eyelid) and ulcerative dermatitis

Cutaneous lesions predominate in the clinical picture of FeL due to *L. infantum*. Dermal abnormalities include nodules, ulcerations or more rarely exfoliative dermatitis. They are generalized or localized, symmetrical or asymmetric and may, though less frequently, appear all over the body in a focal, multifocal, regional or diffuse pattern [12–14, 26, 36, 37, 51, 52, 56, 58, 60, 62, 64, 68, 70]. Some cats may harbour different types of skin lesions at the same time or develop them subsequently; they may coexist with mucocutaneous lesions (Fig. 3). Cutaneous and mucocutaneous nodules, of variable size, are more often localized on the head, including eyelids, nose and lips, or on the distal parts of the limbs. Nodules have also been reported in the anal mucosa [68] and they are usually small (less than 1 cm), non painful or pruritic and have a normal, ulcerated or alopecic surface [26, 50, 51, 56, 60, 62–64, 66, 68, 70].

**Fig. 4:**
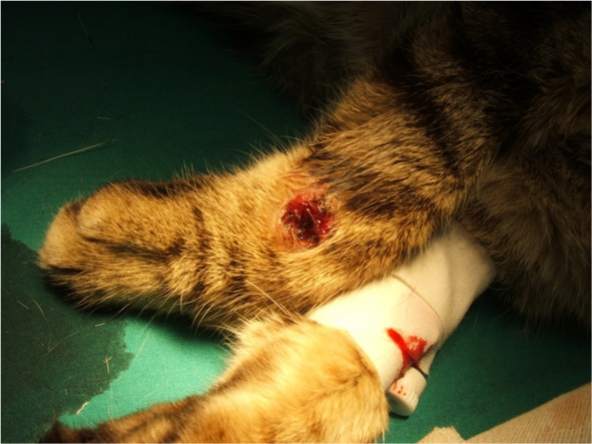
Clinical findings of feline leishmaniosis due to *Leishmania infantum*: ulcerative dermatitis on distal limb

Ulcerations which may be diffuse and superficial or focal and deep (Fig. 4) are localized on the same body sites as nodules, and may be complicated by bacterial infections that explain why they are covered by hemorrhagic crusts and/or purulent material [13, 14, 52, 53, 56, 58, 60–62, 64, 65, 68, 70]. However, ulcerative dermatitis is sometimes diffuse and can be observed on the body trunk or on bony prominences [14, 36, 58, 62, 63].

**Fig. 5:**
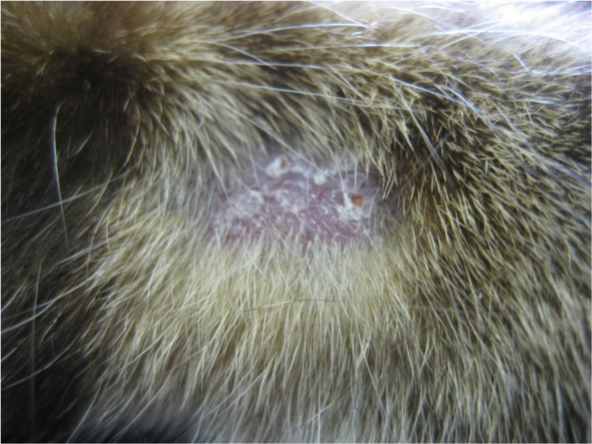
Clinical findings of feline leishmaniosis due to *Leishmania infantum*: focal alopecia and scales

**Fig. 6:**
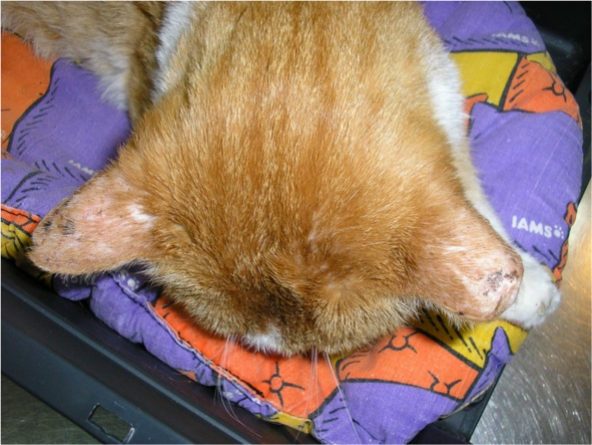
Clinical findings of feline leishmaniosis due to *Leishmania infantum*: symmetrical alopecia on pinnae and acral thickening of the margin of left ear

In contrast to CanL, exfoliative dermatitis (Fig. 5) is rare in the feline disease [36, 52, 68]. Other uncommon dermatologic presentations include hemorrhagic papules and nodules where *Leishmania* amastigotes can be found [37, 52]. Alopecia (Fig. 6), which is also uncommon in FeL [12, 36, 52, 62, 64], may be associated with other skin diseases concurring in *L. infantum* infected cats such as demodicosis [64]. Mild to severe pruritus is rare in FeL [58, 64, 65] and in some cases with a pruritic syndrome other compatible causes co-existed such as flea allergy [52], *pemphigus foliaceus* (PF) [56] or neoplasia (squamous cell carcinoma) [14].

Clinical disease caused by natural infection with species other than *L. infantum* is typically reported as nodular or ulcerative dermatitis with no systemic clinical signs. Skin lesions are often single but they can metastatize (Table 5) [73–76].

**Table 5:** Clinical cases of feline leishmaniosis caused by species other than Leishmania infantum

*Leishmania* species	Geographic location	Signalment	Lesions and outcome	Reference
*L. amazonensis*	Brazil	2-year-old female	Single, nodular lesion (2 cm in diameter) on the nose and many nodules of different sizes on the ears and digital regions; smears from lesion aspirates with numerous amastigotes. Respiratory failure and euthanasia some days after diagnosis	[73]
*L. braziliensis*	Brazil	4-year-old female	Cutaneous ulcer (0.5 cm in diameter) present for 6 months on the nose, enlargement of the *planum nasale* and two additional ulcers on the left face (0.3 cm in diameter each). Good general condition. Outcome not reported	[74]
		5-year-old female	Papule on the bridge of the nose and vegetating lesion on the nasal mucosa for 3 months. Good general condition. Outcome not described	
	French Guiana	3 to 5-year-old female	Cutaneous ulcer (1 cm in diameter) on the nose (for ~8 month) and nodules of different sizes on the ears. Outcome not reported	[93]
*L. mexicana*	USA (Texas)	Immunocompetent long-haired adult male followed up for 7 years	Four large (4–7 mm) and many small nodules initially confined to the left ear; lesions with numerous amastigote forms	[75]
			Two years after a radical pinnectomy, the animal had lesion recurrence at the stump, and lesions later developed on the muzzle and nasal mucosa; treatment was attempted several times, but with no resolution	
	USA (Texas)	8 domestic cats (5 males and 3 females) aged 1 to 11 years old (median: 3 years)	One or multiple nodules on the pinnae and less commonly on the muzzle and periorbital skin, with variably ulcerated, scaled or smooth surfaces (histology: numerous amastigotes)	[76]
			Two cats had recurrent cutaneous leishmaniosis: one was treated with allopurinol, but the skin lesions continued to recur despite treatment; in three other cats, excisional biopsy was apparently curative, and lesions did not recur during the follow-up period (2–4 years)	
*L. venezuelensis*	Venezuela (Lara state)	4 cats	One cat: nodular lesion (2 cm) on the nose and six smaller nodules on the ears; two cats: single nodules (2–3 cm) on the nose; one cat: single nodule on the nose (2–3 cm) and 3 months afterwards presented with metastatic new lesions on the ears, tail and lower limbs	[105]

8. What are the most common dermatopathological features of FeL?

Skin histopathology of lesions associated with *L. infantum* has shown that the most commonly observed alteration is a granulomatous dermatitis [26, 51, 56, 59, 60, 68]. It often has a diffuse pattern and the epidermis may present hyperkeratosis, acanthosis and ulceration [56, 68]. A nodular to diffuse arrangement of the granulomatous dermatitis is also reported [26, 60]. However, in a retrospective case series from Spain, two cats presented different histological findings [68]. The first one had granulomatous perifolliculitis with a high number of lymphocytes and plasma cells surrounding the cutaneous adnexa. It was associated with a marked hyperplasia of epidermis and sebaceous glands. The other cat was diagnosed with a lichenoid interface dermatitis typically represented by infiltration of lymphocytes, plasma cells and a few neutrophils and macrophages at the dermoepidermal junction. In this case, epidermal necrosis and epidermal microabscesses were also observed. A perivascular infiltration of superficial skin layers by macrophages, mast cells, neutrophils and eosinophils was also observed in another case [12].

*Leishmania* amastigotes have always been identified in the affected skin. A semiquantitative estimation of amastigotes was also performed with the aid of immunohistochemistry (IHC) [68], in which the parasitic load of the skin ranged from high (>50 immunolabelled amastigotes/field at x400) to moderate (10–50 immunolabelled amastigotes/field) in cases of diffuse granulomatous dermatitis [68]. Conversely, it was low (1–9 immunolabelled amastigotes/field) in cases of granulomatous perifolliculitis or lichenoid interface dermatitis [68] .

In biopsy samples taken from cases with ulcerative dermatitis, eosinophilic granulomatous dermatitis with a severe dermo-epidermal necrosis were found without the presence of amastigotes, but with a positive quantitative *Leishmania* PCR [62].

In some FeL cases, other dermatological diseases such as eosinophilic granuloma and PF were also diagnosed [52, 56, 68].

Interestingly, amastigotes were also found associated with neoplastic tissue in the lesion of two cats with squamous cell carcinoma (SCC) [13]. In one other case, SCC was diagnosed in a cat presenting concurrent *Leishmania* skin lesions [14, 59].

In two cases of skin disease caused by *L. braziliensis*, a mononuclear and neutrophilic inflammatory infiltrate of the dermal tissue was seen in histological sections [77].

9. What are the most common differential diagnoses in *L. infantum* endemic areas for dermatological features?

The commonly seen cutaneous nodular form in FeL cases should be distinguished from nodules caused in cats with cryptococcosis, sporotrichosis, histoplasmosis, sterile or eosinophilic granuloma, mycobacterioses, and a wide variety of cutaneous neoplasms (e.g. feline sarcoids, mast cell tumor, fibrosarcoma, basal cell carcinoma, bowenoid *in situ* carcinoma and lymphoma). The main differentials of the ulcerative lesions include squamous cell carcinoma with which however it may co-exist [13, 14, 59], idiopathic ulcerative dermatitis, indolent ulcer, mosquito-bite dermatitis, atypical mycobacteriosis and feline leprosy, cutaneous vasculitis, erythema multiforme and cold-agglutinin disease. Finally, skin diseases such as dermatophytosis, systemic or cutaneous lupus erythematosus, exfoliative dermatitis due to thymoma or due to immune-mediated pathomecanisms, PF, sebaceous adenitis/mural folliculitis complex and paraneoplastic alopecia could be included in the differential list of those leishmanial cats that are admitted with the rare exfoliative/crusting dermatitis which may also be alopecic and erythematous. It has been postulated that PF and FeL may share a common pathomechanism (molecular mimicry) when they co-exist in the same cat [56].

10. What clinicopathological findings may alert the clinician to the possibility of FeL due to *L. infantum*?

Limited information is available about clinicopathological abnormalities in cats and it is only based on case reports. Mild to severe normocytic normochromic non-regenerative anemia is the most frequent haematological abnormality reported in clinical cases [37]. Moderate to severe pancytopenia may be observed [37, 50, 57] in association with aplastic bone marrow, but some of the cats reported with pancytopenia were FIV positive [37, 50, 57]. Curiously, in one of these cases, amastigotes were found in 4 % of neutrophils in buffy coat smears [57].

Hyperproteinemia with hypergammaglobulinemia is a common finding in FeL as also found in dogs [2], and hypoalbuminemia is occasionally reported [37, 50].

Renal proteinuria and increased serum creatinine are also reported at diagnosis or during follow-up in some cases [37, 68].

Relative lymphocytosis and an increase in serum ALT activity were significantly associated with seroreactivity to *L. infantum* [78].

**Fig. 7:**
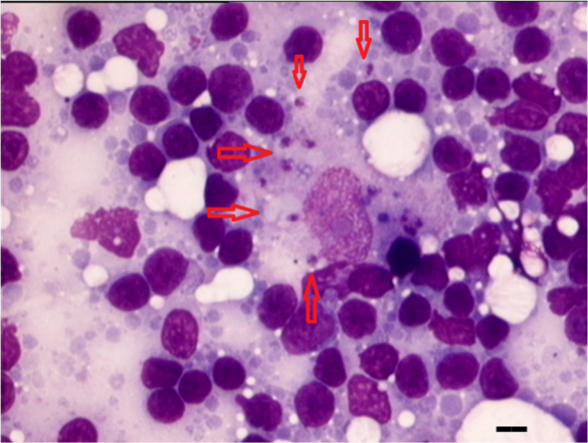
Fine-needle aspirate of a reactive lymph node from a cat with feline leishmaniosis due to *Leishmania infantum*: lymphoid hyperplasia and a macrophage with *L. infantum* amastigotes (red arrows). May-Grünwald-Giemsa stain, scale bar = 20 μm

The type of inflammatory infiltrate found in tissue cytology (aspirates, impression smears) or histopathology in organs such as skin, eye, oral mucosa, liver, spleen and kidney is commonly pyogranulomatous to granulomatous [66, 68, 72]. There was also lymphoid reactive hyperplasia in lymphoid organs such as lymph nodes [79] and spleen [57], with variable numbers of *Leishmania* amastigotes observed (Fig. 7).

11. What are the most common differential diagnoses in endemic areas for systemic illness caused by *L. infantum* in cats?

As lymph node enlargement is the most common sign, apart from skin and mucocutaneous lesions, FeL should be included in the differential list when this finding is noted on physical examination as solitary or generalized lymphadenomegaly. This list mainly includes infections with other infectious agents (FIV, FeLV, FCoV, *Bartonella*, *Mycobacteria*, *T. gondii*, *Cryptococcus* or other systemic mycoses), lymphoma or metastatic involvement from other neoplasia.

FeL should also be considered in cats with ophthalmologic disease, mainly in cats with acute, recurring or chronic uveitis and differentiated from similar clinical conditions caused by FIV, FeLV, FCoV, *Bartonella, T. gondii*, fungal infections, neoplasia or paraneoplastic syndrome. Some feline uveitis cases are considered idiopatic and treated with corticosteroids. A diagnosis of idiopatic uveitis was initially made in some cases of ocular FeL and corticosteroids worsened the disease [50, 55, 69]. This fact warrants a careful investigation to exclude FeL before treating ocular disease with corticosteroids.

Proliferative and ulcerative chronic inflammation of the oral mucosa associated with FeL can be included in the list of possible causes of the feline chronic gingivostomatitis syndrome (FCGS). This painful and common immune-mediated disease is considered multifactorial in cats and treated by full mouth teeth extraction for eliminating oral plaque antigenic stimulation. Corticosteroids are frequently used to improve the clinical signs; however, when this was tried in some cats with oral disease associated with *L. infantum* infection it induced worsening of FeL [11, 66].

Hyperglobulinemia with increased gammaglobulin level reported in FeL is usually found in chronic infections caused by viruses, bacteria or systemic fungi, or inflammation associated with FCGS or inflammatory bowel disease, or in neoplasia such as lymphoma, or multiple myeloma.

### Diagnosis

12. On what tests should the evaluation of *L. infantum* infection be based in cats with suspected clinical leishmaniosis?

Most diagnostic techniques for *Leishmania* infection which are available for dogs are also employed in cats. Diagnosis is made in the majority of cases by serologic, cytologic, histologic, culture or PCR methods (Table 6).

**Table 6:** Laboratory methods for diagnosis of Leishmania infection in cats

Method	Principle	Features	Recommendations	References
Serology	Detection of specific antibodies by IFAT and ELISA (more frequently used), DAT and WB	Different sensitivities and specificities, partially dependent on the cut-off values; clinical cases may have from low to high positive antibody levels, but the latter are usually diagnostic	Antibodies should be evaluated using techniques validated in cats; parasitological methods should be employed in clinically suspect but seronegative or low positive cats	[23–25, 82–84]
Cytology	Detection of amastigotes in stained tissue smears (ex: lymph node, bone marrow, skin and cornea)	Specific, but time-consuming and requiring expertise	For compatible skin or mucosal lesions, enlarged lymph nodes and other lesions, and for clinically suspected cases if serology is negative or low positive	[50, 61, 63, 65]
Histology with IHC	Detection of amastigotes in histopathology tissue specimens	Specific, but time-consuming and requiring expertise		[59, 68, 69, 72]
		IHC is not widely available		
Culture	Multiplication of promastigotes from tissues	Not suitable for rapid diagnosis and not widely available	For research and species and/or strain identification	[26, 37]
PCR	Amplification of parasite DNA from tissues and biological fluids, including blood, buffy coat, bone marrow, lymph nodes and conjunctival swabs	More sensitive than cytology or histology with IHC; may allow molecular characterization and quantification of the parasitic load	Preferable to sample more than one tissue, in order to increase sensitivity of detection especially in subclinical infections	[24, 66, 69, 97, 98]

DAT: direct agglutination test; ELISA: enzyme-linked immunosorbent assay; IFAT: immunofluorescence antibody test; IHC: immunohistochemistry; PCR: polymerase chain reaction; WB, western blot

The most common serological test used appears to be the immunoflurescence antibody test (IFAT). A validated cut off value of 1:80 has been recommended in cats tested by this serological technique and the serum antibody level to *Leishmania* antigen ranged from low to high positive levels in clinical cases of FeL [24]. Quantitative enzyme-linked immunosorbent assays (ELISA) are also frequently employed and seems to be more sensitive than IFAT [80–82]. The direct agglutination test (DAT) was found less sensitive than IFAT [27] or ELISA [23] and western blot (WB) was more sensitive than IFAT [83].

Clinical cases of FeL with positive sera have specific antibodies against *L. infantum* antigens of low molecular mass (≤31 kDa) [12, 22] by WB. These low molecular mass antigens are considered to be also the most specific polypeptides in the diagnosis of human [84, 85] and CanL [86, 87]. It is important to highlight that cats from both endemic and non endemic areas may be positive against high molecular weight antigens. This is also observed in dogs and humans and it is considered as a cross-reaction probably due to the presence of antibodies to the heat shock protein 70 family [22].

In general, anti-*Leishmania* antibodies should always be evaluated by laboratories using serological methods validated in cats.

Cross-reactions exist between feline antibodies to different *Leishmania* and *Trypanosoma* species as also shown in dogs, but they do not seem to occur with antibodies to *T. gondii* [28, 66].

Amastigotes were found in blood smears and smears from nasal exudate or corneal cytology [50, 57, 63, 66].

The diagnostic procedure in cats positive to *Leishmania* infection should always be completed with specific tests for excluding other compatible or concurrent diseases.

13. Should healthy cats or cats under specific conditions be tested for *L. infantum* infection?

*Leishmania infantum* can infect apparently healthy cats, and as with dogs, infection may persist with no clinical manifestations [88]. Since cats infected with *L. infantum* may not be sick and, therefore, not present any clinical signs, it is questionable whether healthy cats should be tested for this infection. In our opinion, cats with no clinical signs and/or clinicopathological abnormalities compatible with leishmaniosis should be tested for *Leishmania* infection if they are used as blood donors, since it has been shown for humans and dogs that blood products from infected individuals may transmit infection [89]. Antibody testing and blood PCR are advisable as indicated for dogs. Furthermore, testing can be done for exportation purposes to countries where leishmaniosis is not endemic and may require cats to be tested for infection before importation. Finally, cats with clinical conditions requiring immunosuppressive therapies should be preliminarily tested in endemic areas, as clinical cases of FeL were diagnosed in cats under long term immunosuppressive treatment.

### Treatment and monitoring

14. What is the most effective specific treatment and the expected clinical response to treatment of FeL due to *L. infantum*?

The published information on the treatment of FeL is extremely limited because it is available from only 20 case reports and only some of them were followed up (Table 7). Allopurinol is the most frequently used drug followed by meglumine antimoniate, but information is lacking on pharmacokinetic and pharmacodynamic characteristics of these drugs in cats and also about their safety.

**Table 7:** Therapeutic regimens used in cats affected by feline leishmaniosis

Drug and dosage	Duration	Number of treated cats	References
Allopurinol (10–15 mg/kg/12 h, 20 mg/kg/24 h, 25 mg/cat/12 h, 100 mg/cat/24 h) PO	6 months - 3 years	15	[14, 37, 50, 54–56, 64, 65]
Meglumine antimoniate (20–50 mg/kg/24 h SC)	20–30 days	1	[59, 63]
Meglumine antimoniate (175 mg/cat/48 h IM)	55 days	1	[51]
Meglumine antimoniate (5 mg/kg/24 h SC) in combination with Ketoconazole (10 mg/kg/24 h PO)	3 cycles of 4 weeks, 10 days apart	1	[36]
Fluconazole (5 mg/kg/24 h PO)	60 days	1^a^	[37]
Spiramycin (150.000 IU/kg) and Metrodinazole (25 mg/kg) 24 h PO	35 days	1^a^	[37]
Itraconazole (50 mg/cat/24 h PO)	60 days	1^a^	[37]

SC: subcutaneous; IM: intamuscular; PO: *per os* ^a^ a same cat was treated with the three different therapeutic regimens at subsequent times

Allopurinol is generally well tolerated; however, in one cat, elevation of hepatic enzymes was reported at 10 mg/kg BID and the dose was reduced to 5 mg/kg BID [56]. Clinical improvement was observed in most cases treated with allopurinol – even in FIV positive cats –within a few weeks after treatment was initated [37, 50, 64] or slowly after 6 months [56]. A long term follow-up was available in some cats treated with allopurinol. A clinical cure was obtained in these cats but relapse occurred after discontinuation of treatment, suggesting that they were still infected [14, 37, 55]. Clinical worsening leading to euthanasia occurred in a few cases after a few weeks of therapy [54, 57].

Clinical cure was generally obtained in the few cats that were treated with meglumine antimoniate, but long term follow up are not available from these cases.

Some other oral drugs (fluconazole, itraconazole, metronidazole and spiramycin) administered to one cat at different times were considered as not effective [37].

Surgical removal of cutaneous nodules (performed in two cats) was followed by relapsing of cutaneous lesions [36, 51].

In conclusion, currently, no scientific evidence concerning the best treatment for FeL is available, but more extensive clinical experience is available for treatment with allopurinol (10 mg/kg BID or 20 mg/kg SID). The drug of choice to be used in FeL should nevertheless be based on the best compliance and safety for the cat with the alternatives of long term oral drug treatment (allopurinol) or a parenteral therapy (meglumine antimoniate). As there are no studies on the safety of these drugs in cats, it is recommended to strictly monitor the health status of animals under treatment by means of regular check-ups including urinalysis, and advising the owner to promptly report any abnormality.

The duration of allopurinol treatment should be evaluated case by case based on clinical response and on parasitological and serological monitoring.

### Prognosis

15. What is the prognosis of clinical leishmaniosis ?

Some consideration can be extrapolated from information reported on 14 cats affected by FeL and followed up until death or euthanasia. On the basis of these reported cases, prognosis appears to vary from good to poor. In fact, five cats died a few days or weeks after diagnosis [12, 26, 36, 37, 65]. Some were affected by chronic renal failure or hepatic disease, but the real influence of *Leishmania* infection on mortality was not clearly demonstrated in these cases [36, 37, 65]. In other cases, euthanasia was performed after diagnosis because of a rapid clinical worsening [54, 57, 62] or due to a concurrent neoplasia [13]. *Post mortem* evaluation was obtained in three cats that died or were euthanized shortly after diagnosis, and all of them had visceral dissemination of *Leishmania* amastigotes found in the spleen, lymph nodes, liver, stomach or in the large bowel [13, 36, 57].

Records of a long-term follow up (13–60 months) are available for nine cats and in four of the cases they were followed up until death or euthanasia [11, 37, 50, 56, 60, 66, 69, 70]. Their age ranged between 5 and 12 years at diagnosis and only one had been found positive for FIV antibodies. Clinical presentation varied but visceral dissemination of *Leishmania* infection was investigated and confirmed in all but one case. This latter cat had a diagnosis of PF associated with *Leishmania* infection confirmed by serology and PCR on skin biopsies, but the potential extra-cutaneous dissemination of infection was not investigated [56]. Four of these followed up cats were treated with allopurinol for 24–40 months [37, 50, 56, 66].

It is noteworthy that three cats which were never treated with anti-*Leishmania* drugs after diagnosis died or were euthanized 1–5 years later and one was reported alive after 4 years. In these untreated cases, FeL progressed with time and chronic renal disease developed in two cats that were not treated. Untreated ocular FeL may cause vision loss and may require ocular enucleation due to panophthalmitis [50, 53, 55, 68, 69].

The retrospective evaluation of single case reports did not provide clear evidence about the prognosis of FeL because the clinical data available are heterogeneous and sometimes incomplete; however, some conclusions can be inferred. Both treated and untreated cats may live for years before the deterioration of their health status mainly due to renal and heart injuries that might be unrelated to *L.infantum* infection. The exact role of *L. infantum* infection in the development of multiorgan injury causing renal, cardiac or hepatic disease has to be confirmed. However, it can significantly influence life expectancy and any concurrent diseases should be treated if detected. In case of renal disease, the International Renal Interest Society (IRIS) staging system is recommended for therapy, follow-up and prognosis (http://www.iris-kidney.com).

### Prevention

16. Can *Leishmania* infection be prevented in cats?

There are two main reasons for employing preventive measures against *L. infantum* infection in a susceptible animal host and suspected reservoir such as the cat: 1) to protect the single animal from the risk of developing a clinical disease; 2) and to contribute to the reduction of the prevalence of infection in a geographic area. However, it should be also pointed out that the epidemiological role of the cat as a main reservoir for *Leishmania* species has not been confirmed [34].

Due to the absence of studies on vaccines against *Leishmania* in cats, the best strategy to prevent *Leishmania* infection in this animal could be to use topical insecticides with application of chemical compounds with sand fly repellent activity, similar to those used for dogs. Unfortunately, most pyrethroids, like permethrin and deltamethrin, cannot be used in cats due to their toxicity to this species. The recent launch of a collar containing an additional compound belonging to this chemical class, flumethrin, that is well tolerated in the cat might represent a valid preventive option for the individual reduction of risk for infection of cats in highly endemic areas of leishmaniosis, and for limiting the infectiousness of those that are already infected. In fact, this collar was found useful in reduction of the incidence of *L. infantum* infection in dogs [90, 91].

## Conclusions

Although the data on FeL supported by consolidated evidence-based studies are limited, these guidelines constitute a baseline for educating and informing feline practitioners with the most comprehensive and updated data set on this important neglected feline protozoal disease.

Further studies need to elucidate gaps in knowledge on this infection in cats and to provide evidence-based information on the management of this disease.

## Abbreviations

ALT: alanine aminotransferase
BID: *bis in die* (twice a day)
CanL: canine leishmaniosis
DAT: direct agglutination test
ELISA: enzyme-linked immunosorbent assay
FCGS: feline chronic gingivostomatitis syndrome
FeL: feline leishmaniosis
FeLV: feline leukemia virus
FCoV: feline coronavirus
FIV: feline immunodeficiency virus
IFAT: immunofluorescence antibody test
IHAT: indirect haemagglutination test
IHC: immunohistochemistry
ILMA: immunolabelling with monoclonal antibodies
IRIS: international renal interest society
MLEE: multilocus isoenzyme electrophoresis
PF: *pemphigus foliaceus*
PCR: polymerase chain reaction
RFLP: restriction fragment length polymorphism
SID: *semel in die* (once a day)
WB: western blot

## References

[1] Solano-Gallego L, Miró G, Koutinas A, Cardoso L, Pennisi MG, Ferrer L (2011). LeishVet guidelines for the practical management of canine leishmaniosis. Parasit Vectors.

[2] Solano-Gallego L, Koutinas A, Miró G, Cardoso L, Pennisi MG, Ferrer L (2009). Directions for the diagnosis, clinical staging, treatment and prevention of canine leishmaniosis. Vet Parasitol.

[3] Sergent E, Sergent E, Lombard J, Quilichini M (1912). La leishmaniose à Alger. Infection simultanée d’un enfant, d’un chien et d’un chat dans la même habitation. Bull Soc Pathol Exot.

[4] Morsy TA, el Seoud SM A (1994). Natural infection in two pet cats in a house of a zoonotic cutaneous leishmaniasis patient in Imbaba area, Giza Governorate, Egypt. J Egypt Soc Parasitol.

[5] Giordano A (1933). Le chat dans la transmission de la leishmaniose viscérale de la méditérranée. Bull Sez Ital Soc Internaz Microbiol.

[6] Morsy TA, Michael SA, Makhlouf LM, el Sibai MM (1988). *Leishmania* infection sought in non human hosts in Suez Governorate, Egypt. J Egypt Soc Parasitol.

[7] Morsy TA, Michael SA, El Disi AM (1980). Cats as reservoir hosts of human parasites in Amman, Jordan. J Egypt Soc Parasitol.

[8] Roudebush P, Allen TA, Dodd CE, Novotny BJ (2004). Application of evidence-based medicine to veterinary clinical nutrition. J Am Vet Med Assoc.

[9] Baneth G, Koutinas AF, Solano-Gallego L, Bourdeau P, Ferrer L (2008). Canine leishmaniosis - new concepts and insights on an expanding zoonosis: part one. Trends Parasitol.

[10] Gramiccia M, Di Muccio T, Vitale F, Caracappa S, Reale S, Pennisi MG. *Leishmania infantum* characterization from three cases of feline leishmaniasis in Sicily (Italy). In: Abstract Book of Worldleish3. Palermo-Terrasini; 2005. p. 146.

[11] Maroli M, Pennisi MG, Di Muccio T, Khoury C, Gradoni L, Gramiccia M (2007). Infection of sandflies by a cat naturally infected with *Leishmania infantum*. Vet Parasitol.

[12] Ozon C, Marty P, Pratlong F, Breton C, Blein M, Lelievre A (1998). Disseminated feline leishmaniosis due to *Leishmania infantum* in Southern France. Vet Parasitol.

[13] Grevot A, Jaussaud Hugues P, Marty P, Pratlong F, Ozon C, Haas P (2005). Leishmaniosis due to *Leishmania infantum* in a FIV and FeLV positive cat with a squamous cell carcinoma diagnosed with histological, serological and isoenzymatic methods. Parasite.

[14] Pocholle E, Reyes-Gomez E, Giacomo A, Delaunay P, Hasseine L, Marty P (2012). Un cas de leishmaniose féline disseminée dans le sud de la France. Parasite.

[15] Baum M, Ribeiro MC, Lorosa ES, Damasio GA, Castro EA (2013). Eclectic feeding behavior of *Lutzomyia* (*Nyssomyia*) *intermedia* (Diptera, Psychodidae, Phlebotominae) in the transmission area of American cutaneous leishmaniasis, state of Parana, Brazil. Rev Soc Bras Med Trop.

[16] Afonso MM, Duarte R, Miranda JC, Caranha L, Rangel EF (2012). Studies on the feeding habits of *Lutzomyia* (*Lutzomyia*) *longipalpis* (Lutz & Neiva, 1912) (Diptera: Psychodidae: Phlebotominae) populations from endemic areas of American visceral leishmaniasis in northeastern Brazil. J Trop Med.

[17] Maroli M, Jalouk L, Al Ahmed M, Bianchi R, Bongiorno G, Khoury C (2009). Aspects of the bionomics of *Phlebotomus sergenti* sandflies from an endemic area of anthroponotic cutaneous leishmaniasis in Aleppo Governorate, Syria. Med Vet Entomol.

[18] Ogusuku E, Perez JE, Paz L, Nieto E, Monje J, Guerra H (1994). Identification of bloodmeal sources of *Lutzomyia* spp. in Peru. Ann Trop Med Parasitol.

[19] Johnson RN, Ngumbi PM, Mwanyumba JP, Roberts CR (1993). Host feeding preference of *Phlebotomus guggisbergi*, a vector of *Leishmania tropica* in Kenya. Med Vet Entomol.

[20] da Silva SM, Rabelo PF, Gontijo Nde F, Ribeiro RR, Melo MN, Ribeiro VM (2010). First report of infection of *Lutzomyia longipalpis* by *Leishmania* (*Leishmania*) *infantum* from a naturally infected cat of Brazil. Vet Parasitol.

[21] Pennisi MG, Hartmann K, Lloret A, Addie D, Belak S, Boucraut-Baralon C (2013). Leishmaniosis in cats: ABCD guidelines on prevention and management. J Feline Med Surg.

[22] Solano-Gallego L, Rodríguez-Cortés A, Iniesta L, Quintana J, Pastor J, Espada Y (2007). Cross-sectional serosurvey of feline leishmaniasis in ecoregions around the Northwestern Mediterranean. Am J Trop Med Hyg.

[23] Cardoso L, Lopes AP, Sherry K, Schallig H, Solano-Gallego L (2010). Low seroprevalence of *Leishmania infantum* infection in cats from northern Portugal based on DAT and ELISA. Vet Parasitol.

[24] Pennisi MG, Lupo T, Malara D, Masucci M, Migliazzo A, Lombardo G (2012). Serological and molecular prevalence of *Leishmania infantum* infection in cats from Southern Italy. J Feline Med Surg.

[25] Sherry K, Miró G, Trotta M, Miranda C, Montoya A, Espinosa C (2011). A serological and molecular study of *Leishmania infantum* infection in cats from the Island of Ibiza (Spain). Vector Borne Zoonotic Dis.

[26] Poli A, Abramo F, Barsotti P, Leva S, Gramiccia M, Ludovisi A (2002). Feline leishmaniosis due to *Leishmania infantum* in Italy. Vet Parasitol.

[27] Sarkari B, Hatam GR, Adnani SJ, Asgari Q (2009). Seroprevalence of feline leishmaniasis in areas of Iran where *Leishmania infantum* is endemic. Ann Trop Med Parasitol.

[28] Longoni SS, López-Cespedes A, Sánchez-Moreno M, Bolio-Gonzalez ME, Sauri-Arceo CH, Rodríguez-Vivas RI (2012). Detection of different *Leishmania* spp. and *Trypanosoma cruzi* antibodies in cats from the Yucatan Peninsula (Mexico) using an iron superoxide dismutase excreted as antigen. Comp Immunol Microbiol Infect Dis.

[29] Tabar MD, Altet L, Francino O, Sanchez A, Ferrer L, Roura X (2008). Vector-borne infections in cats: molecular study in Barcelona area (Spain). Vet Parasitol.

[30] Nasereddin A, Salant H, Abdeen Z (2008). Feline leishmaniasis in Jerusalem: serological investigation. Vet Parasitol.

[31] Sobrinho LS, Rossi CN, Vides JP, Braga ET, Gomes AA, de Lima VM (2012). Coinfection of *Leishmania chagasi* with *Toxoplasma gondii*, Feline Immunodeficiency Virus (FIV) and Feline Leukemia Virus (FeLV) in cats from an endemic area of zoonotic visceral leishmaniasis. Vet Parasitol.

[32] Pennisi MG, Maxia L, Vitale F, Masucci M, Borruto G, Caracappa S (2000). Studio dell’infezione da *Leishmania* mediante PCR in gatti che vivono in zona endemica. Atti Soc Ital Sci Vet.

[33] Ayllon T, Diniz PP, Breitschwerdt EB, Villaescusa A, Rodriguez-Franco F, Sainz A (2012). Vector-borne diseases in client-owned and stray cats from Madrid, Spain. Vector Borne Zoonotic Dis.

[34] Miró G, Rupérez C, Checa R, Gálvez R, Hernández L, García M (2014). Current status of *L. infantum* infection in stray cats in the Madrid region (Spain): implications for the recent outbreak of human leishmaniosis?. Parasit Vectors.

[35] Pennisi MG, Masucci M, Catarsini O (1998). Presenza di anticorpi anti-*Leishmania* in gatti FIV+ che vivono in zona endemica. Atti Soc Ital Sci Vet.

[36] Hervás J, Chacón-M De Lara F, Sánchez-Isarria MA, Pellicer S, Carrasco L, Castillo JA (1999). Two cases of feline visceral and cutaneous leishmaniosis in Spain. J Feline Med Surg.

[37] Pennisi MG, Venza M, Reale S, Vitale F, Lo Giudice S (2004). Case report of feline leishmaniasis in four cats. Vet Res Comm.

[38] Quinnell RJ, Courtenay O (2009). Transmission, reservoir hosts and control of zoonotic visceral leishmaniasis. Parasitology.

[39] Kirkpatrick CE, Farrell JP, Goldschmidt MH (1984). *Leishmania chagasi* and *L. donovani*: experimental infections in domestic cats. Exp Parasitol.

[40] Millan J, Ferroglio E, Solano-Gallego L (2014). Role of wildlife in the epidemiology of *Leishmania infantum* infection in Europe. Parasitol Res.

[41] Molina R, Jiménez MI, Cruz I, Iriso A, Martín-Martín I, Sevillano O (2012). The hare (*Lepus granatensis*) as potential sylvatic reservoir of *Leishmania infantum* in Spain. Vet Parasitol.

[42] Moreno I, Álvarez J, García N, de la Fuente S, Martínez I, Mariño E (2014). Detection of anti-*Leishmania infantum* antibodies in sylvatic lagomorphs from an epidemic area of Madrid using the indirect immunofluorescence antibody test. Vet Parasitol.

[43] Maia C, Campino L (2011). Can domestic cats be considered reservoir hosts of zoonotic leishmaniasis?. Trends Parasitol.

[44] Pennisi MG (2015). Leishmaniosis of companion animals in Europe: an update. Vet Parasitol.

[45] Anjili CO, Githure JI (1993). Refractoriness of domestic cats to infection with a Kenyan strain of *Leishmania donovani*. East Afr Med J.

[46] Simões-Mattos L, Mattos MR, Teixeira MJ, Oliveira-Lima JW, Bevilaqua CM, Prata-Júnior RC (2005). The susceptibility of domestic cats (*Felis catus*) to experimental infection with *Leishmania braziliensis*. Vet Parasitol.

[47] Moreno J, Alvar J (2002). Canine leishmaniasis: epidemiological risk and the experimental model. Trends Parasitol.

[48] Maia C, Nunes M, Cristóvão J, Campino L (2010). Experimental canine leishmaniasis: clinical, parasitological and serological follow-up. Acta Trop.

[49] Figueredo LA, de Paiva-Cavalcanti M, Almeida EL, Brandão-Filho SP, Dantas-Torres F (2012). Clinical and hematological findings in *Leishmania braziliensis*-infected dogs from Pernambuco, Brazil. Rev Bras Parasitol Vet.

[50] Richter M, Schaarschmidt-Kiener D, Krudewig C (2014). Ocular signs, diagnosis and long-term treatment with allopurinol in a cat with leishmaniasis. Schweiz Arch Tierheilkd.

[51] Costa Durão JFC, Rebelo E, Peleteiro MC, Correia JJ (1994). Primeiro caso de leishmaniose em gato doméstico (*Felis catus*) detectado em Portugal (Concelho de Sesimbra). Nota preliminar. Rev Port Cienc Vet.

[52] Laurelle-Magalon C, Toga I (1996). Un cas de leishmaniose féline. Prat Med Chir Anim Comp.

[53] Hervás J, Chácon-Manrique de Lara F, López J, Gómez-Villamandos JC, Guerrero MJ, Moreno A (2001). Granulomatous (pseudotumoral) iridociclitis associated with leishmaniasis in a cat. Vet Rec.

[54] Britti D, Vita S, Aste A, Williams DA, Boari A (2005). Sindrome da malassorbimento in un gatto con leishmaniosi. Atti Soc Ital Sci Vet.

[55] Leiva M, Lloret A, Pena T, Roura X (2005). Therapy of ocular and visceral leishmaniasis in a cat. Vet Ophthalmol.

[56] Rüfenacht S, Sager H, Müller N, Schaerer V, Heier A, Welle MM (2005). Two cases of feline leishmaniosis in Switzerland. Vet Rec.

[57] Marcos R, Santos M, Malhão F, Pereira R, Fernandes AC, Montenegro L (2009). Pancytopenia in a cat with visceral leishmaniasis. Vet Clin Pathol.

[58] Dunan N, Mary C, Garbe L, Breton Y, Olivon B, Ferrey P (1989). A propos d’un cas de leishmaniose chez un chat de la région marseillaise. Bull Soc Fr Parasitol.

[59] Monteverde V, Polizzi D, Lupo T, Fratello A, Leone C, Buffa F, et al. Descrizione di un carcinoma a cellule squamose in corso di leishmaniosi in un gatto. In: Proceedings of the 7th National Congress of the Italian Society of Veterinary Laboratory Diagnostics (SIDiLV). Perugia; 2006. p. 329–30.

[60] Ortuñez A, Gomez P, Verde MT, Mayans L, Villa D, Navarro L. Lesiones granulomatosas en la mucosa oral y lengua y muliples nodulos cutaneos en un gato causado por *Leishmania infantum*. In: Proceedings of the Southern European Veterinary Conference. Barcelona; 2010.

[61] Pennisi MG, Venza M (1999). Case report of *Leishmania* spp. infection in two cats from the Aeolian archipelago (Italy). Proceedings of the 24th WSAVA Congress.

[62] Dalmau A, Ossò M, Oliva A, Anglada L, Sarobé X, Vives E (2008). Leishmaniosis felina a propósito de un caso clínico. ¿Nos olvidamos de que existe?. Clin Vet Peq Anim.

[63] Ibba F (2009). Un caso di rinite cronica in corso di leishmaniosi felina. Proceedings of the 62nd International SCIVAC Congress.

[64] Sanches A, Pereira AG, Carvalho JP (2011). Um caso de leishmaniose felina. Vet Med.

[65] Ennas F, Calderone S, Caprì A, Pennisi MG (2012). Un caso di leishmaniosi felina in Sardegna. Veterinaria.

[66] Migliazzo A, Vitale F, Calderone S, Puleio R, Binanti D, Abramo F (2015). Feline leishmaniosis: a case with a high parasitic burden. Vet Dermatol.

[67] Caracappa S, Migliazzo A, Lupo T, Lo Dico M, Calderone S, Rea S, et al. Analisi biomolecolari, sierologiche ed isolamento in un gatto infetto da *Leishmania* spp. In: Proceedings of the 7th National Congress of the Italian Society of Veterinary Laboratory Diagnostics (SIDiLV). Alghero; 2008. p. 134–5.

[68] Navarro JA, Sánchez J, Peñafiel-Verdú C, Buendía AJ, Altimira J, Vilafranca M (2010). Histopathological lesions in 15 cats with leishmaniosis. J Comp Pathol.

[69] Verneuil M (2013). Leishmaniose oculaire féline: à propos d’un cas. J Fr Ophtalmol.

[70] Pennisi MG, Lupo T, Migliazzo A, Persichetti MF, Masucci M, Vitale F. Feline leishmaniosis in Italy: retrospective evaluation of 24 clinical cases. In: Abstract Book of the 5th World Congress on Leishmaniasis. Porto de Galinhas; 2013. P837.

[71] Hervás-Rodríguez J, Pérez-Arévalo J, Chacón-M de Lara F, López Fernández J, Moreno Boiso A, Gómez-Villamandos J (2002). Evaluation of local immunoresponse in feline leishmaniasis. Proceedings of the 27th WSAVA Congress.

[72] Puleio R, Tamburello A, Lupo T, Migliazzo A, Loria GR, Pennisi MG. Aspetti istopatologici, immunoistochimici e molecolari in quattro casi di leishmaniosi felina. In: Proceedings of the 8th National Congress of the Italian Assocation of Veterinary Pathology (AIPVet). Padua; 2011.

[73] de Souza AI, Barros EM, Ishikawa E, Ilha IM, Marin GR, Nunes VL (2005). Feline leishmaniasis due to *Leishmania* (*Leishmania*) *amazonensis* in Mato Grosso do Sul State, Brazil. Vet Parasitol.

[74] Schubach TM, Figueiredo FB, Pereira SA, Madeira MF, Santos IB, Andrade MV (2004). American cutaneous leishmaniasis in two cats from Rio de Janeiro, Brazil: first report of natural infection with *Leishmania* (*Viannia*) *braziliensis*. Trans R Soc Trop Med Hyg.

[75] Barnes JC, Stanley O, Craig TM (1993). Diffuse cutaneous leishmaniasis in a cat. J Am Vet Med Assoc.

[76] Trainor KE, Porter BF, Logan KS, Hoffman RJ, Snowden KF (2010). Eight cases of feline cutaneous leishmaniasis in Texas. Vet Pathol.

[77] Veronesi F, Moretta I, Vitale F, Lupo T, Migliazzo A, Mariani C, et al. *Leishmania infantum*: serological and molecular investigation in cats from Ischia island. In: Proceedings of the 2nd International Congress on Canine Leishmaniasis. Pisa; 2010. p. 169–71.

[78] Ayllon T, Tesouro MA, Amusategui I, Villaescusa A, Rodriguez-Franco F, Sainz A (2008). Serologic and molecular evaluation of *Leishmania infantum* in cats from Central Spain. Ann N Y Acad Sci.

[79] Perillo L, Pennisi MG, Solano-Gallego L, Lupo T, Migliazzo A, Mazzullo G (2013). *Leishmania infantum* PCR positive lymph node aspirates: cytologic patterns in cats. Proceedings of the International SCIVAC Congress “Canine leishmaniasis and other vector-borne diseases: our current state of knowledge”.

[80] Figueiredo FB, Bonna IC, Nascimento LD, da Costa T, Baptista C, Pacheco TM (2009). Avaliação sorológica para detecção de anticorpos anti-*Leishmania* em cães e gatos no bairro de Santa Rita de Cássia, Município de Barra Mansa, Estado do Rio de Janeiro. Rev Soc Bras Med Trop.

[81] Coelho WM, do Amarante AF, Apolinário J de C, Coelho NM, de Lima VM, Perri SH (2011). Seroepidemiology of *Toxoplasma gondii*, *Neospora caninum*, and *Leishmania* spp. infections and risk factors for cats from Brazil. Parasitol Res.

[82] da Silveira Neto L, Sobrinho LS, Martins CO, Machado RZ, Marcondes M, de Lima VM (2011). Use of crude, FML and rK39 antigens in ELISA to detect anti-*Leishmania* spp. antibodies in *Felis catus*. Vet Parasitol.

[83] Marechal M (1993). La leishmaniose feline: cas sporadique ou realité encore ignorée?.

[84] Mary C, Lamouroux D, Dunan S, Quilici M (1992). Western blot analysis of antibodies to *Leishmania infantum* antigens: potential of the 14-kD and 16-kD antigens for diagnosis and epidemiologic purposes. Am J Trop Med Hyg.

[85] Riera C, Fisa R, López-Chejade P, Serra T, Girona E, Jiménez M (2008). Asymptomatic infection by *Leishmania infantum* in blood donors from the Balearic Islands (Spain). Transfusion.

[86] Aisa MJ, Castillejo S, Gallego M, Fisa R, Riera MC, de Colmenares M (1998). Diagnostic potential of Western blot analysis of sera from dogs with leishmaniasis in endemic areas and significance of the pattern. Am J Trop Med Hyg.

[87] Iniesta L, Gállego M, Portús M (2007). Idiotype expression of IgG1 and IgG2 in dogs naturally infected with *Leishmania infantum*. Vet Immunol Immunopathol.

[88] Chatzis MK, Leontides L, Athanasiou LV, Papadopoulos E, Kasabalis D, Mylonakis M (2014). Evaluation of indirect immunofluorescence antibody test and enzyme-linked immunosorbent assay for the diagnosis of infection by *Leishmania infantum* in clinically normal and sick cats. Exp Parasitol.

[89] Owens SD, Oakley DA, Marryott K, Hatchett W, Walton R, Nolan TJ (2001). Transmission of visceral leishmaniasis through blood transfusions from infected English foxhounds to anemic dogs. J Am Vet Med Assoc.

[90] Otranto D, Dantas-Torres F, de Caprariis D, Di Paola G, Tarallo VD, Latrofa MS (2013). Prevention of canine leishmaniosis in a hyper-endemic area using a combination of 10 % imidacloprid/4.5 % flumethrin. PLoS One.

[91] Brianti E, Gaglio G, Napoli E, Falsone L, Prudente C, Solari Basano F (2014). Efficacy of a slow-release imidacloprid (10 %)/flumethrin (4.5 %) collar for the prevention of canine leishmaniosis. Parasit Vectors.

[92] Passos VM, Lasmar EB, Gontijo CM, Fernandes O, Degrave W (1996). Natural infection of a domestic cat (*Felis domesticus*) with *Leishmania* (*Viannia*) in the metropolitan region of Belo Horizonte, State of Minas Gerais, Brazil. Mem Inst Oswaldo Cruz.

[93] Rougeron V, Catzeflis F, Hide M, De Meeus T, Banuls AL (2011). First clinical case of cutaneous leishmaniasis due to *Leishmania* (*Viannia*) *braziliensis* in a domestic cat from French Guiana. Vet Parasitol.

[94] Hatam GR, Adnani SJ, Asgari Q, Fallah E, Motazedian MH, Sadjjadi SM (2010). First report of natural infection in cats with *Leishmania infantum* in Iran. Vector Borne Zoonotic Dis.

[95] Millán J, Zanet S, Gomis M, Trisciuoglio A, Negre N, Ferroglio E (2011). An investigation into alternative reservoirs of canine leishmaniasis on the endemic island of Mallorca (Spain). Transbound Emerg Dis.

[96] Maia C, Nunes M, Campino L (2008). Importance of cats in zoonotic leishmaniasis in Portugal. Vector Borne Zoonotic Dis.

[97] Maia C, Gomes J, Cristóvão J, Nunes M, Martins A, Rebelo E (2010). Feline *Leishmania* infection in a canine leishmaniasis endemic region, Portugal. Vet Parasitol.

[98] Maia C, Ramos C, Coimbra M, Bastos F, Martins A, Pinto P (2014). Bacterial and protozoal agents of feline vector-borne diseases in domestic and stray cats from southern Portugal. Parasit Vectors.

[99] Chatzis MK, Andreadou M, Leontides L, Kasabalis D, Mylonakis M, Koutinas AF (2014). Cytological and molecular detection of *Leishmania infantum* in different tissues of clinically normal and sick cats. Vet Parasitol.

[100] Savani ES, de Oliveira Camargo MC, de Carvalho MR, Zampieri RA, dos Santos MG, D’Auria SR (2004). The first record in the Americas of an autochthonous case of *Leishmania* (*Leishmania*) *infantum chagasi* in a domestic cat (*Felix catus*) from Cotia County, São Paulo State, Brazil. Vet Parasitol.

[101] da Silva AV, de Souza Cândido CD, de Pita Pereira D, Brazil RP, Carreira JC (2008). The first record of American visceral leishmaniasis in domestic cats from Rio de Janeiro, Brazil. Acta Trop.

[102] Coelho WM, Richini-Pereira VB, Langoni H, Bresciani KD (2011). Molecular detection of *Leishmania* sp. in cats (*Felis catus*) from Andradina Municipality, São Paulo State, Brazil. Vet Parasitol.

[103] Coelho WM, Lima VM, Amarante AF, Langoni H, Pereira VB, Abdelnour A (2010). Occurrence of *Leishmania* (*Leishmania*) *chagasi* in a domestic cat (*Felis catus*) in Andradina, São Paulo, Brazil: case report. Rev Bras Parasitol Vet.

[104] Vides JP, Schwardt TF, Sobrinho LS, Marinho M, Laurenti MD, Biondo AW (2011). *Leishmania chagasi* infection in cats with dermatologic lesions from an endemic area of visceral leishmaniosis in Brazil. Vet Parasitol.

[105] Bonfante-Garrido R, Urdaneta I, Urdaneta R, Alvarado J (1991). Natural infection of cats with *Leishmania* in Barquisimeto, Venezuela. Trans R Soc Trop Med Hyg.

[106] Michael SA, Morsy TA, El-Seoud SF, Saleh MS (1982). Leishmaniasis antibodies in stray cats in Ismailiya Governorate, Egypt. J Egypt Soc Parasitol.

[107] Diakou A, Papadopoulos E, Lazarides K (2009). Specific anti-*Leishmania* spp. antibodies in stray cats in Greece. J Feline Med Surg.

[108] Huebner J, Müller E, Langbein-Detsch I, Naucke T, Kissingen B (2008). Serological survey of *Leishmania* infections in cats from north Greece. Proceedings of ACVIM Forum.

[109] Silaghi C, Knaus M, Rapti D, Kusi I, Shukullari E, Hamel D (2014). Survey of *Toxoplasma gondii* and *Neospora caninum*, haemotropic mycoplasmas and other arthropod-borne pathogens in cats from Albania. Parasit Vectors.

[110] Vita S, Santori D, Aguzzi I, Petrotta E, Luciani A (2005). Feline leishmaniasis and ehrlichiosis: serological investigation in Abruzzo region. Vet Res Commun.

[111] Spada E, Proverbio D, Migliazzo A, Della Pepa A, Perego R, Bagnagatti De Giorgi G (2013). Serological and molecular evaluation of *Leishmania infantum* infection in stray cats in a nonendemic area in northern Italy. ISRN Parasitol.

[112] Portús M, Gállego M, Riera C, Aisa MJ, Fisa R, Castillejo S (2002). Wild and domestic mammals in the life cycle of *Leishmania infantum* in Southwest Europe. A literature review and studies performed in Catalonia (Spain). Rev Iber Parasitol.

[113] Zárate-Ramos JJ, Arbea-Sarasa I, Gómez-Ochoa P, Castillo-Hernández JA, García-Salinas MJ, Morales-Amella MJ (2002). Serological evidence of leishmaniasis in cats in Aragon, Spain. Proceedings of the 27th WSAVA Congress.

[114] Martín-Sánchez J, Acedo C, Muñoz-Pérez M, Pesson B, Marchal O, Morillas-Márquez F (2007). Infection by *Leishmania infantum* in cats: epidemiological study in Spain. Vet Parasitol.

[115] Miró G, Hernández L, Montoya A, Arranz-Solís D, Dado D, Rojo-Montejo S (2011). First description of naturally acquired *Tritrichomonas foetus* infection in a Persian cattery in Spain. Parasitol Res.

[116] Duarte A, Castro I, da Fonseca IM P, Almeida V, de Carvalho LM M, Meireles J (2010). Survey of infectious and parasitic diseases in stray cats at the Lisbon Metropolitan Area, Portugal. J Feline Med Surg.

[117] Vilhena H, Martinez-Díaz VL, Cardoso L, Vieira L, Altet L, Francino O (2013). Feline vector-borne pathogens in the north and centre of Portugal. Parasit Vectors.

[118] Maia C, Ramos C, Coimbra M, Cardoso L, Campino L (2014). Prevalence of *Dirofilaria immitis* antigen and antibodies to *Leishmania infantum* in cats from southern Portugal. Parasitol Int.

